# Using Graphene-Based Materials for Stiff and Strong Poly(ethylene glycol) Hydrogels

**DOI:** 10.3390/ijms23042312

**Published:** 2022-02-19

**Authors:** Helena P. Ferreira, Duarte Moura, Andreia T. Pereira, Patrícia C. Henriques, Cristina C. Barrias, Fernão D. Magalhães, Inês C. Gonçalves

**Affiliations:** 1 i3S—Instituto de Investigação e Inovação em Saúde, Universidade do Porto, Rua Alfredo Allen 208, 4200-135 Porto, Portugal; helena.ferreira@i3s.up.pt (H.P.F.); duarte.moura@i3s.up.pt (D.M.); andreia.pereira@i3s.up.pt (A.T.P.); ap.henriques@ineb.up.pt (P.C.H.); ccbarrias@ineb.up.pt (C.C.B.); 2 INEB—Instituto de Engenharia Biomédica, Universidade do Porto, Rua Alfredo Allen 208, 4200-135 Porto, Portugal; 3 ICBAS—Instituto de Ciências Biomédicas Abel Salazar, Universidade do Porto, Rua Jorge de Viterbo Ferreira 228, 4050-313 Porto, Portugal; 4 FEUP—Faculdade de Engenharia, Departamento de Engenharia Metalúrgica e de Materiais, Universidade do Porto, Rua Dr. Roberto Frias, 4200-465 Porto, Portugal; 5 LEPABE—Laboratório de Engenharia de Processos, Ambiente, Biotecnologia e Energia, Faculdade de Engenharia da Universidade do Porto, Rua Dr. Roberto Frias, 4200-465 Porto, Portugal; fdmagalh@fe.up.pt

**Keywords:** biomaterials, PEG, graphene oxide, tensile strength, anti-adhesive surface, cardiovascular applications

## Abstract

Blood-contacting devices are increasingly important for the management of cardiovascular diseases. Poly(ethylene glycol) (PEG) hydrogels represent one of the most explored hydrogels to date. However, they are mechanically weak, which prevents their use in load-bearing biomedical applications (e.g., vascular grafts, cardiac valves). Graphene and its derivatives, which have outstanding mechanical properties, a very high specific surface area, and good compatibility with many polymer matrices, are promising candidates to solve this challenge. In this work, we propose the use of graphene-based materials as nanofillers for mechanical reinforcement of PEG hydrogels, and we obtain composites that are stiffer and stronger than, and as anti-adhesive as, neat PEG hydrogels. Results show that single-layer and few-layer graphene oxide can strengthen PEG hydrogels, increasing their stiffness up to 6-fold and their strength 14-fold upon incorporation of 4% *w/v* (40 mg/mL) graphene oxide. The composites are cytocompatible and remain anti-adhesive towards endothelial cells, human platelets and *Staphylococcus aureus*, similar to neat hydrogels. To the best of our knowledge, this is the first work to report such an increase of the tensile properties of PEG hydrogels using graphene-based materials as fillers. This work paves the way for the exploitation of PEG hydrogels as a backbone material for load-bearing applications.

## 1. Introduction

Cardiovascular diseases (CVD) are the number one cause of death worldwide, and, every year, approximately 17.9 million people die from it, according to the World Health Organization [1]. CVD include various conditions, namely coronary or peripheral artery diseases, cerebrovascular disease (stroke), valvular diseases, heart failure and venous thromboembolism [2]. Most of these are managed through alterations to lifestyle, medication, minimally-invasive interventions or surgery. Blood-contacting devices (BCD) are an increasingly important resource for the management of some CVD, including prosthetic heart valves (for valvular diseases), stents and vascular grafts (for coronary and peripheral artery disease) and ventricular assist devices (for treatment of heart failure), among others [3]. The combined global market of these BCD is estimated at USD 32 billion and is predicted to grow in the coming years [4,5,6,7]. 

Although BCD vary greatly in terms of composition, application and duration of use, there is one common aspect among them: they must ensure normal blood circulation. Despite being a topic of intense research for the past 20 years, long-term hemocompatibility still poses a challenge for BCD, and they continue to face issues of thrombogenicity, immunogenicity and/or infection. For example, Dacron vascular grafts fail due to thrombus formation in 52% of above-knee bypasses performed in peripheral artery disease patients [8,9]. Thrombosis occurs in up to 5.7% of surgically-placed mechanical heart valves [10,11]. In addition, infection remains a persistent problem to be solved. After heart failure, infection is the leading cause of death related to left ventricular assist device implantation, accounting for 16% of these deaths with an incidence of 17.46 infections per 100 patient-months [12,13]. 

BCD-associated thrombosis is essentially triggered by plasma protein adsorption on the device’s surface. Adsorbed fibrinogen, even at extremely low concentrations, promotes platelet adhesion, activation and aggregation. Thrombin converts fibrinogen into fibrin, which interacts with platelet aggregates to form a thrombus [14]. Thus, the development of biomaterials to prevent thrombus formation is key for overcoming BCD failure. 

Poly(ethylene glycol) (PEG) hydrogels have been explored for various biomedical applications, given their high water-retention capacity, biocompatibility and non-fouling properties [15]. They are also biologically “inert” and can thus serve as a “blank slate” for specific functionalization, providing highly tunable physicochemical and biological properties for the intended application. Although some studies have used PEG-based polymers as coatings [16,17], this approach presents limitations, particularly due to inadequate coating-substrate binding [18]. To overcome these problems, it would be valuable to explore PEG hydrogels as a bulk material for the fabrication of BCD. This has been explored in only a couple of papers in the literature [19,20], likely because the weak mechanical properties of PEG hydrogels render them unsuitable for this purpose. Strategies to toughen hydrogels include the use of interpenetrating polymer networks, double network structures and the incorporation of micro/nanofillers [21].

Graphene is a single-layer honeycomb lattice of sp^2^-bonded carbon atoms with outstanding stability, mechanical and electroconductive properties [22]. Since its isolation in 2004, graphene-based materials (GBM) have been explored for several applications, particularly for purposes of mechanical, thermal and electroconductive enhancement. GBM include materials which may vary in lateral size (diagonal size of planar sheet), thickness (number of layers) and oxidation degree (number of oxygen-containing groups) [23]. Oxidized forms, in particular, are proven to be cytocompatible [24]. Additionally, studies show that GBM-containing surfaces and polymers have antimicrobial effects [23,25]. Thus, GBM appear to be interesting fillers for improvement of the mechanical properties of hydrogels used in biomedical applications [26,27]. 

There are only a few papers in the literature that explore PEG/GBM composite hydrogels (Supplementary Information (SI), Table S1) [28,29,30,31,32,33,34]. Overall, graphene oxide (GO) is the most explored GBM, with the highest reported concentration being 1.2% *w/v* (12 mg/mL). Most of these works focus on the characterization of compressive or rheological properties, while little is shown about tensile properties. A greater focus on this aspect is essential for application of these composite hydrogels as bulk materials in devices subjected to tensile stress, such as BCD. Xiao and colleagues reported an 8-fold increase in the tensile strength of polyvinyl alcohol/PEG (6 kDa) hydrogels with 1.2% *w/v* GO (12 mg/mL), corresponding to the greatest mechanical reinforcement reported for PEG/GBM composite hydrogels [30]. Jang and coworkers reported the stiffest and strongest formulation of this type, corresponding to a PEG (2 kDa) hydrogel with 0.4% *w/v* (4 mg/mL) GO, via compressive Young’s modulus and compressive strength of 40 and 1000 kPa, respectively [29]. Importantly, the non-fouling and anti-adhesive properties of these hydrogels have been vastly overlooked.

In this work, we seek to fill these gaps by exploring different GBM as nanofillers for the reinforcement of PEG hydrogels and by characterizing their tensile and physicochemical properties. Envisioning the potential application of these composite hydrogels in BCD, we have also assessed their anti-adhesive behavior towards endothelial cells, blood platelets and bacteria. 

## 2. Results

### 2.1. FLGO and FLG Platelets Are Larger than GO 

**Figure 1:**
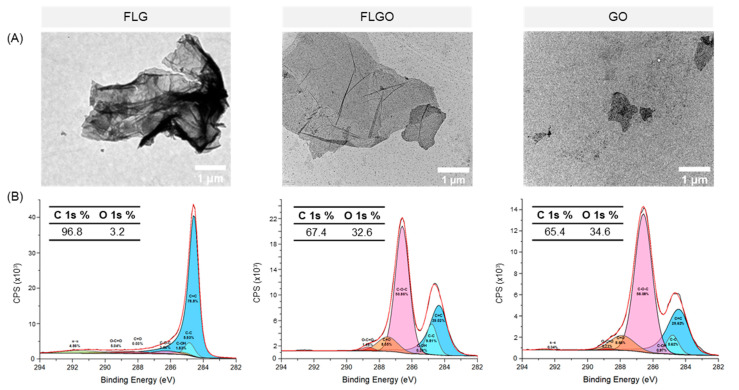
FLG, FLGO and GO characterization. (**A**) TEM images of GBM dispersions in water: scale bar = 1 μm. (**B**) High-resolution C 1s spectra of GBM; percentage of C 1s and O 1s determined from overall survey spectra; and percentage of C=C, C-C, C-OH, C-O-C, C=O, O-C=O and π-π bonds determined by deconvolution of high-resolution spectra.

GBM of different thicknesses, lateral sizes and oxidation degrees were explored as nanofillers for the mechanical reinforcement of PEG hydrogels, particularly reduced forms—few-layer graphene (FLG)—and oxidized forms—graphene oxide (GO) and few-layer graphene oxide (FLGO). These materials were characterized in terms of size and morphology by transmission electron microscopy (TEM) and chemical composition by X-ray photoelectron spectroscopy (XPS) (Figure 1). TEM images show that the FLG and FLGO particles had a higher lateral size, while the GO particles were smaller and mostly appeared single-layer, as expected. Also, the FLG platelets seemed to present some agglomeration, probably due to the lack of oxygen-containing groups, which diminishes their interaction with the aqueous media (Figure 1A). According to XPS analysis (Figure 1B), FLG had a 3.2% oxidation degree, while FLGO and GO had similar oxidation degrees of 32.6% and 34.6%, respectively, confirming the successful oxidation of FLG and graphite by modified Hummers’ method, respectively. A high-resolution C 1s spectrum of FLG revealed that C=C sp2 bonds were the main chemical group present (85.1%), as would be expected for a reduced GBM. In FLGO and GO, the main oxygen-containing groups were epoxides (C-O-C) and carbonyls (C=O). Hence, these GBM had distinct sizes and/or oxidation degrees. FLG and FLGO can be compared to understand the influence of oxidation degree on the properties of PEG/GBM composite hydrogels. GO and FLGO can be compared to understand the effect of morphology (lateral size and thickness).

### 2.2. Oxidized GBM can Mechanically Reinforce PEG Hydrogels

Depending on the type of tissue or its application, BCD can be subjected to multiple mechanical stresses. In particular, vascular grafts and heart valves are mainly subjected to surface shear and tensile stresses caused by blood flow. Thus, the tensile properties of biomaterials for application in BCD should be characterized. Additionally, most papers on graphene-reinforced PEG hydrogels report characterization of compressive or rheological properties, i.e., there is a gap in the literature regarding the tensile properties of these composite materials.

**Figure 2:**
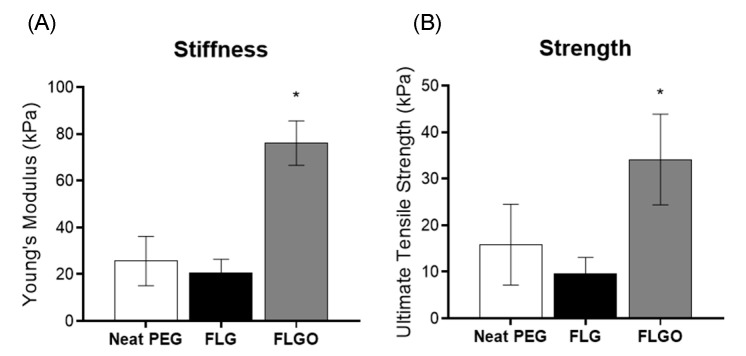
Effect of GBM oxidation degree on tensile properties of PEG/GBM composite hydrogels. (**A**) YM and (**B**) UTS of neat PEG hydrogels, 1% FLG and 1% FLGO composite hydrogels. One-way ANOVA, Tukey’s multiple comparisons test, * *p* < 0.05.

The tensile properties of neat PEG and PEG/GBM composite hydrogel films were herein evaluated according to ASTM Standard D882-02 2002 [35]. The effect of GBM parameters (oxidation degree, size and concentration) on the tensile properties of PEG/GBM composite hydrogels were assessed first. For the effect of oxidation degree, the tensile properties of composites with 1% *w/v* FLG or 1% *w/v* FLGO were compared. Results show that the incorporation of 1% *w/v* FLG did not reinforce PEG hydrogels (Figure 2), while the same loading of FLGO yielded a 196% increase in stiffness and 115% in tensile strength. As such, further studies focused on oxidized forms only (i.e., GO and FLGO).

**Figure 3:**
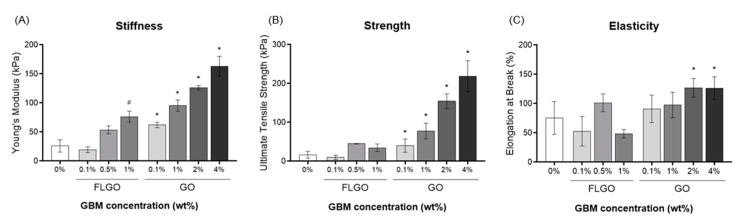
Effect of GBM morphology on tensile properties of PEG/GBM composite hydrogels. (**A**) YM, (**B**) UTS and (**C**) EB of neat PEG hydrogels, 0.1, 0.5 and 1% FLGO and 0.1, 1, 2 and 4% GO composite hydrogels. At least two technical replicates and two independent experiments for FLGO composites: Kruskal–Wallis analysis, Dunn’s multiple comparisons test, # *p*< 0.05 vs. neat PEG hydrogels. Five technical replicates and two independent experiment for GO composites: one-way ANOVA, Tukey’s multiple comparisons test, * *p* < 0.05 vs. neat PEG hydrogels.

To evaluate the effect of morphology (i.e., lateral size and thickness) and concentration, composite hydrogels with either FLGO (0.1, 0.5 or 1% *w/v*) or GO (0.1, 1, 2, or 4% *w/v*) were produced, and tensile properties were tested (Figure 3). The maximum possible concentrations for dispersion were 1% *w/v* FLGO and 4% *w/v* GO, since mixtures with higher amounts of GBM became highly viscous and difficult to homogenize, degas and handle. 5% GO composite hydrogels are presented in SI (Figure S1), showing the presence of air bubbles as well as big GO aggregates in the hydrogel matrix and that their strength and elasticity was not higher than that of 4% GO composites. Therefore, the maximum concentration explored for further experiments was 4% GO. 

The Young’s moduli (YM) (Figure 3A) showed that composites with 1% FLGO or ≥0.1% GO were significantly stiffer than neat PEG hydrogels. The PEG/1% FLGO composite hydrogels presented a YM of 76 ± 9.5 kPa, and the PEG/4% GO had a YM of 163 ± 17 kPa, which were, respectively, 3-fold and 6-fold higher than the YM of the neat PEG hydrogels (26 ± 11 kPa). Regarding the ultimate tensile strength (UTS) (Figure 3B), stronger hydrogels were only obtained via the incorporation of GO, with 4% GO composite hydrogels reaching 218 ± 40 kPa, which is 14-fold higher than the neat PEG hydrogels (16 ± 8.7 kPa). Elongation at break (EB) (Figure 3C) in the FLGO composites appeared to be unrelated to concentration, although the decrease with 1% FLGO should be noticed. Alternatively, in the GO composites, EB tended to increase with GO concentration; and the PEG/4% GO composites reached up to 126 ± 16% of the initial size. Overall, the oxidized forms of graphene (GO and FLGO) were shown to mechanically reinforce PEG hydrogels. In particular, 4% GO provided the best tensile properties, increasing not only the stiffness and strength, but also the elasticity.

### 2.3. PEG/GBM Composites Maintain Hydrophilicity of Neat PEG Hydrogels

**Figure 4:**
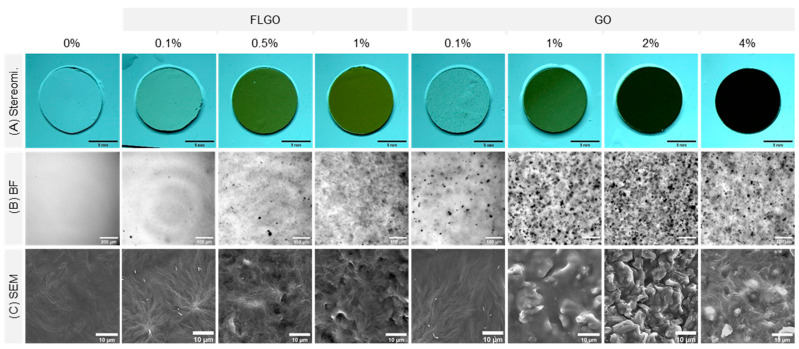
Macroscopic appearance, GBM dispersion in matrix and surface roughness of PEG/GBM composite hydrogels. (**A**) Stereomicroscope images of composite hydrogels; scale bar: 1 mm. (**B**) Brightfield images from widefield microscopy showing GBM dispersion in hydrogel matrix; scale bar: 100 μm. (**C**) SEM images showing surface roughness of composite hydrogels; scale bar: 10 μm.

The GO and FLGO composite hydrogels were further evaluated in terms of macroscopic appearance (stereomicroscopy), GBM dispersion in the matrix (brightfield microscopy) and surface topography (scanning electron microscopy, SEM) (Figure 4). The increase in GBM concentration resulted in hydrogels with darker color (Figure 4A), and a higher number of GBM platelets was also observed throughout the hydrogel matrix. The GO composites appeared to have more aggregates than the FLGO composites (Figure 4B). Regarding surface topography, SEM images showed that the neat PEG hydrogels have smooth surfaces. For the FLGO and GO composites, increasing the filler concentration tended to lead to an increase in surface roughness (Figure 4C).

**Figure 5:**
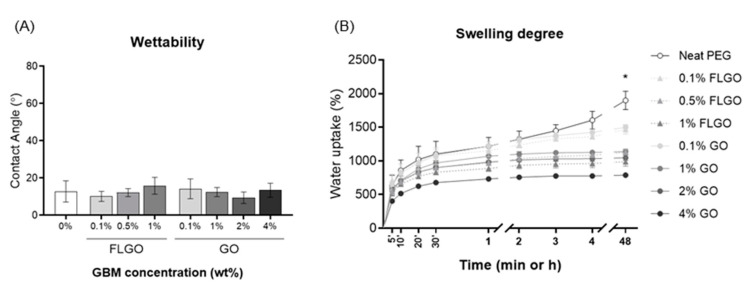
Wettability and swelling degree of PEG/GBM composite hydrogels. (**A**) Water contact angle of hydrated composite films. Five technical replicates and two independent experiments: one-way ANOVA and Tukey’s multiple comparisons test; no statistically significant differences found. (**B**) Swelling degree of pre-dried hydrogels at different timepoints (5, 10, 20, 30 min, 1, 2, 3, 4 and 48 h). Five technical replicates and two independent experiments: one-way ANOVA and Tukey’s multiple comparisons test; * *p* < 0.05 vs. neat PEG hydrogels.

The water contact angle of the hydrated hydrogels was evaluated using the inverted air drop method (Figure 5A). There were no significant differences between the neat PEG hydrogels and the FLGO or GO composites, and all conditions presented a contact angle of <20°. Therefore, GBM incorporation did not alter the hydrophilicity of the PEG hydrogels, which remained highly hydrophilic. The swelling degree of neat and composite hydrogels was evaluated by measuring the water uptake of dried samples and was higher in the neat PEG hydrogels (around 1900% vs. dried samples). Overall, the higher the GBM concentration, the lower the swelling degree, and, for the same concentration, the FLGO composites swelled slightly less than the GO composites. Even so, the swelling degree remained high (>780%) for all conditions (Figure 5B).

### 2.4. Extracts of PEG/GBM Composite Hydrogels Are Cytocompatible towards HUVEC

The cytocompatibility of the neat PEG and PEG/GBM composite hydrogels’ extracts was tested using a human umbilical vein endothelial cell (HUVEC) line, a relevant cell type for biomaterials that contact with blood.

In order to confirm that neat and composite hydrogels did not produce cytotoxic leachables or degradation products, extracts of these biomaterials were prepared in culture medium for 24 h, according to ISO10993-12 [36], and their cytocompatibility towards HUVEC was evaluated according to ISO10993-05 [37]. 

**Figure 6:**
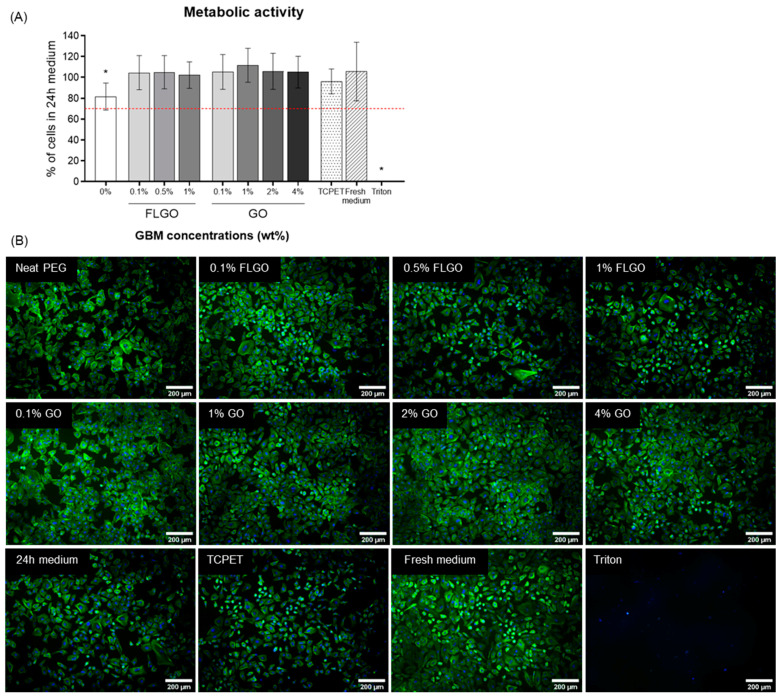
Cytocompatibility of PEG/GBM composite hydrogels’ extracts. (**A**) Metabolic activity, expressed in relation to 24 h medium (positive control of cell viability), of HUVEC incubated for 24 h with extracts of neat PEG, GO and FLGO composites, TCPET (reference material), fresh medium and 0.1% Triton X-100 (negative control). Five technical replicates and two independent experiments: one-way ANOVA and Tukey’s multiple comparisons test, * *p* < 0.05. (**B**) Fluorescence images of HUVEC morphology after incubation with extracts from the aforementioned materials; actin filaments stained with phalloidin (green) and nuclei counterstained with DAPI (blue); scale bar: 200 μm.

Resazurin results show that, for all FLGO and GO composites, the HUVEC metabolic activity was around 100% relative to the positive control (cells cultured with 24 h medium) and similar to reference material (TCPET extracts) (Figure 6A). Interestingly, the neat PEG hydrogels’ extracts caused some decrease in metabolic activity (though still higher than 70%, which is the conventional threshold established for cytocompatibility). This could be related to the higher swelling degree of neat PEG hydrogels, allowing for release of more leachables (despite thorough washing performed after gelation). Also, fluorescence microscopy images showed that HUVEC cultured with the hydrogels’ extracts presented similar size and morphology and reached a similar confluency state, as compared to cells cultured with 24 h medium (control condition) and fresh medium (Figure 6B).

### 2.5. Neither HUVEC, Platelets or Bacteria Adhere onto the Surface of PEG/GBM Composite Hydrogels

**Figure 7:**
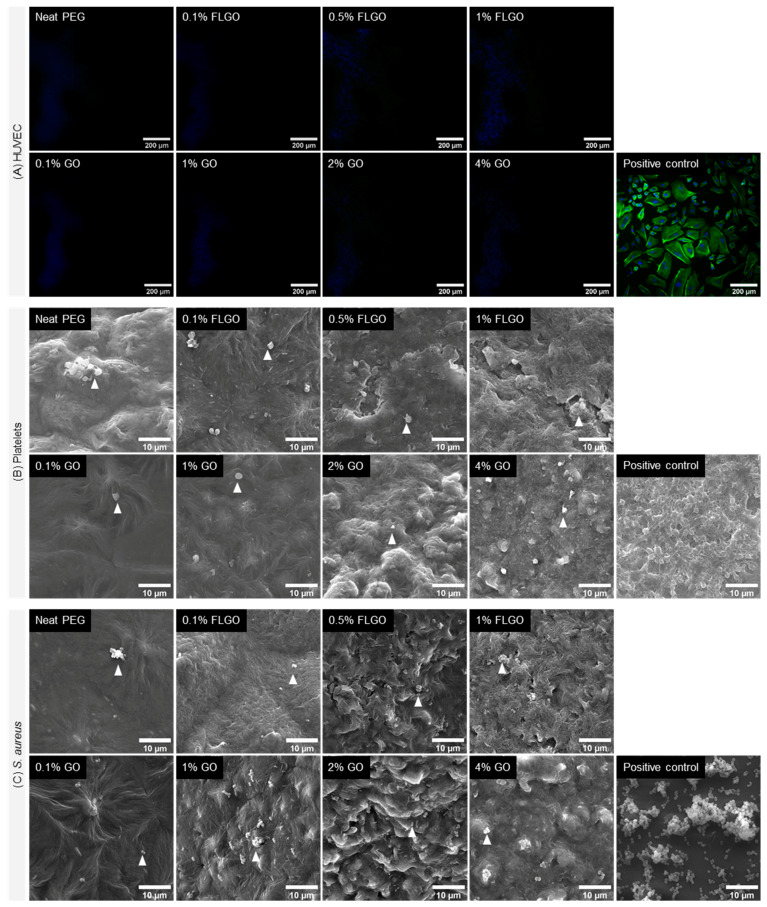
Anti-adhesiveness of PEG/GBM composite hydrogels. (**A**) Fluorescence images of HUVEC adhesion on neat PEG, GO and FLGO composites and gelatin-coated TCPS wells (positive control); actin filaments stained with phalloidin (green) and nuclei counterstained with DAPI (blue); scale bar: 200 μm. (**B**) SEM images of platelets adhered on neat PEG, GO and FLGO composites and glass (positive control); arrowheads indicate platelets; scale bar: 10 μm. (**C**) SEM images of *S. aureus* adhered on neat PEG, GO and FLGO composites and TCPET (positive control); arrowheads indicate bacteria; scale bar: 10 μm.

To probe whether the incorporation of GBM altered the typical antiadhesive properties of PEG hydrogels, materials were challenged toward HUVEC, platelets and bacteria (Figure 7).

HUVEC were seeded directly on top of hydrogels. Fluorescence microscopy images of Day 7 reveal that there were practically no cells adhered to the surface of either the neat PEG, PEG/GO or PEG/FLGO composite hydrogels (Figure 7A). (Further confirmed by the low metabolic activity on samples from Days 1 and 7 of culture, shown in SI, Figure S2).

Platelet adhesion and activation was verified by incubation of samples with platelet concentrates for 1 h. SEM images show that both neat and composite surfaces had very few platelets adhered, and these were mainly in some hydrogel defects. Furthermore, adherent platelets presented a round morphology, indicating that the surface of composite hydrogels did not lead to platelet activation (Figure 7B) [38]. 

Bacterial adhesion to PEG and PEG/GBM composite hydrogels was assessed by incubating these materials with *S. aureus*, the most common strain in BCD infection. SEM images indicate that both neat and composite hydrogels had very few bacteria adhered to the surface, unlike the positive control, where biofilm-like structures could already be found (Figure 7C).

## 3. Discussion

There is a considerable gap in the literature regarding the incorporation of GBM in PEG hydrogels. A comprehensive characterization of mechanical (particularly tensile) and biological properties is an important step in exploring these composite hydrogels and demonstrating their potential for blood-contacting devices, which are subjected to tensile loads (e.g., vascular grafts, cardiac valves). 

In order to evaluate the effect of oxidation degree of GBM in the mechanical reinforcement of PEG hydrogels, we chose to compare FLG and FLGO (which presented 3.2% and 32.6% oxidation degree, respectively, and equivalent platelet sizes). It is theoretically conceivable that FLG is stronger than FLGO, given the integrity of the sp^2^-bond structure in the planar sheets (similar to graphene vs. GO [39]). However, we observed that the incorporation of FLG had no effect on the tensile properties of the PEG hydrogels. This is probably due to the lack of oxygen-containing groups in FLG, which prevents its adequate dispersion within the aqueous hydrogel mixture and interaction with the PEG chains. On the contrary, FLGO and GO establish hydrogen bonds and van der Waals forces with PEG chains, allowing for interfacial stress transfer during deformation. As such, these results show that the presence of oxygen-containing groups in the GBM nanofillers improve their interfacial interaction with the polymeric network, leading to a mechanical reinforcement of the hydrogel [40]. This observation is in line with previous works focused on PEG hydrogels (SI, Table S1), where most authors explored the incorporation of GO and report a mechanical improvement under compression, and Pal et al. show a decrease in compressive modulus when using graphene materials with lower oxidation degree [33]. To the best of our knowledge, this is the first work, in PEG hydrogels, to unequivocally demonstrate the impact of this parameter (oxidation) by direct comparison of GBM with different oxidation degrees. 

Regarding the oxidized GBM (GO and FLGO), the maximum concentrations that were possible to homogeneously disperse in PEG were 1% *w/v* FLGO and 4% *w/v* GO. This is the highest concentration of any GBM to be incorporated in PEG hydrogels ever reported in the literature [30], providing a reference range of concentrations for future works. The tested oxidized GBM differ in terms of both lateral size (FLGO sheets are larger than GO) and thickness (GO is exfoliated to become single-layer, unlike FLGO). Even though FLGO has a larger lateral size, GO is more exfoliated, therefore providing a larger specific surface area (surface area *per* unit mass) for stress transfer. Hence, for the same concentration of nanofiller added (0.1 or 1% *w/v*), mechanical reinforcement is more effective with GO, due to its particles being better exfoliated than those of FLGO. Furthermore, it is important to notice the reinforcement is not linearly proportional to the GO concentration. This may be explained by the formation of GO agglomerates in the PEG matrix (as seen in the bright field images of GBM dispersion in matrix): although concentration increases, it does not translate into a proportionally larger nanofillers’ interfacial area. Altogether, these results support the importance of efficient exfoliation of GBM nanofillers for better reinforcement of hydrogels [41]. 

In neat PEG hydrogels, increasing the crosslinking density (through lower molecular weight PEG) typically increases YM and UTS and decreases EB [42]. However, in GO-containing composites, the improvement in stiffness and strength appeared not to significantly affect elasticity. In fact, the opposite was shown: YM, UTS and EB tended to increase with higher GO concentrations. This had previously been reported for other GBM-containing composites [26,43]. The tuning of elasticity may be useful for the application of these composite hydrogels in BCD, which should be stiff/strong enough to withstand various mechanical stresses but must also support deformation and be easily handled and implanted. 

Direct comparisons of the mechanical properties of different PEG/GBM composite hydrogels described in the literature should be conducted with caution, due to differences in formulations (PEG structure, molecular weight and concentrations) and mechanical testing (sample dimensions, testing method and conditions), yet, the results presented herein are in line with those reported. Jang and coworkers, for example, also reported an increase in compressive stiffness and strength, for PEG (6 kDa)/GO composite hydrogels [29].

Other carbon-based materials have also been explored in this context, particularly carbon nanotubes. For example, Van den Broek et al. have shown a 1.6-fold increase in the elastic modulus (under sheer stress and compression) of PEG hydrogels with 0.015% *w/v* of carbon nanotubes (despite working with softer hydrogels, <10 kPa compressive modulus) [44]. Similarly, the authors identified a non-linear relationship between the mechanical improvement of the composites and carbon nanotubes concentration. 

It is important to analyze the mechanical properties of the obtained biomaterials in the context of BCD. To be used as a bulk material, specific tissues/devices should be used as a reference, since mechanical stresses and properties vary greatly depending on the site/goal. Taking heart valves into consideration [45], for example, the elastic moduli of the aortic valve in the radial and circumferential directions are initially around 2–10 kPa and 20–100 kPa [46], and the maximum physiological stress in the aortic valve leaflets has been estimated to be 200–400 kPa [47]. The PEG/4% GO composites have a stiffness and strength within this ranges, suggesting they could be applied in the fabrication of prosthetic heart valves. Another potential application would be vascular grafts. In order to perform a coronary or peripheral artery bypass, cardiovascular surgeons use conduits, which may be autologous vessels (commonly the internal mammary artery or saphenous vein) or synthetic vascular grafts. The mechanical properties of synthetic grafts should resemble those of bypassed vessels or autologous alternatives. The mean circumferential stress of coronary arteries under a physiological blood pressure of 100 mmHg is estimated to be 150 kPa [48]. The elastic modulus of the internal mammary artery under a transmural pressure of 150 mmHg has been reported as 7.7 kPa [49]. Hence, the PEG/4% GO composites could also be considered for the fabrication of vascular grafts.

PEG hydrogels are generally regarded as bioinert and non-degradable [15,50]. Moreover, the non-covalent interactions established between oxidized GBM sheets and PEG chains resulted in a tighter network and lower swelling degree, which may also be indicative of even higher stability. The increase of chemomechanical stability over time caused by GO incorporation has been reported for other hydrogels [51,52]. Therefore, degradation of the polymeric backbone of the hydrogel is not expected to happen, nor is the release of GBM nanoplatelets into the host microenvironment. Altogether, these data indicate that the developed PEG/GO composite hydrogels should be stable over time when implanted as/in a blood-contacting medical device. Extracts from all formulations were cytocompatible, as shown by both the metabolic activity and morphology of HUVEC. This preliminary assay ensures that, when applied in a BCD and under superficial mechanical stress, materials should not release cytotoxic levels of leachables or extracts, at least in the short-term. 

Due to their bioinert and non-fouling character, PEG hydrogels are “blank slates”, i.e., their physicochemical and biological properties can be finely tuned for the desired end via specific modifications. In order to maintain such tunability, the incorporation of GBM should not alter the anti-adhesiveness of neat PEG hydrogels. 

Both the FLGO and GO composites showed hydrophilicity comparable to that of the neat PEG hydrogels with a water contact angle <20°. It has been reported that the FLGO and GO films produced by vacuum filtration present water contact angles of 42° and 33°, respectively [53]. The maintenance of high hydrophilicity in composite hydrogels may be explained by the fact that GBM are encapsulated within the hydrogel matrix, with almost no sheets or sharp edges exposed at the surface (as shown in SEM images). 

When placed in direct contact with either HUVEC, platelets or *S. aureus*, GBM composite hydrogels displayed an overall anti-adhesive behavior. Even though endothelialization may be a suitable strategy to promote hemocompatibility (particularly in heart valves and vascular grafts), it may also compromise the performance of some BCD when uncontrolled (particularly by neointimal hyperplasia in vascular stents and grafts) [54]. By preventing endothelial cell adhesion, PEG/GBM composite hydrogels could be safely applied in those types of BCD without further modifications. Alternatively, they could be tuned to promote adhesion and repopulation with specific cell types in a controlled manner by, for example, grafting PEG macromers with cell-adhesive or protease-sensitive peptide sequences [55,56]. These biomaterials also prevent platelet and *S. aureus* adhesion, which correspond to the onset steps of thrombogenesis and infection, respectively. These features are key for a safe application in any type of BCD, particularly for implantable and long-term devices. 

## 4. Materials & Methods

### 4.1. Synthesis of GBM

Carbon graphite (7–11 μm diameter according to supplier) was purchased from American Elements (California, CA, USA). FLG (xGnP grade M, around 5 μm lateral size according to supplier) was purchased from XG Sciences (Lansing, CA, USA). GO and FLGO were obtained from graphite and FLG, respectively, by modified Hummers’ method [53]. Briefly, 3 g of either graphite or FLG was added to 150 mL of a 4:1 H_2_SO_4_ (Sigma, MO, USA)/H_3_PO_4_ (Thermo Fisher Scientific, Waltham, NJ, USA) mixture and stirred for 10 min in an ice bath (closely controlling the temperature with a thermostat to avoid significant variations). Then, the mixture was cooled to 0 °C using the ice bath. Next, 18 g of KMnO_4_ was gradually added to the mixture, after which the mixture was transferred to a heating plate and kept at 35 °C, under stirring, for 2 h. Then, it was cooled again to 0 °C using an ice bath. An amount of 450 mL of distilled water was then added to the mixture (temperature kept below 40 °C). The mixture was homogenized for 10 min to allow for reaction completion. To eliminate excess KMnO_4_ (Sigma), 20 mL of H_2_O_2_ was slowly added until oxygen release was not visible. The produced material (GO or FLGO) deposited overnight (ON) and was washed in order to remove acidic materials and other contaminants, by centrifuging (4000 rpm, 20 min), decanting the supernatant and adding more water. The washing step was repeated 7–10 times, until the pH of the supernatant matched the pH of distilled water. To obtain GO, the graphite oxide suspension was sonicated for 6 h in an ultrasonic bath (Bondelin Sonorex Digitec, 320 W). FLGO was not sonicated, since exfoliation of the FLG platelets into single sheets was not intended. These GBM were stored as dispersions in water until further use. 

### 4.2. Characterization of GBM

GBM were characterized in terms of size and morphology by transmission electron microscopy (TEM) and oxidation degree by X-ray photoelectron spectroscopy (XPS).

#### 4.2.1. Transmission Electron Microscopy (TEM)

GBM morphology was observed by TEM using a Jeol JEM 1400 TEM (Tokyo, Japan) coupled with a digital CCED Orious 1100W (Tokyo, Japan). GBM (50 μg/mL) was dispersed in Milli-Q type 1 water, sonicated (15 min in ultrasonic bath) and dropped on 200 mesh copper TEM grids.

#### 4.2.2. X-ray Photoelectron Spectroscopy (XPS)

Chemical composition and oxidation degree of GBM were determined by XPS analysis. Measurements were performed at the International Iberian Nanotechnology Laboratory (INL, Braga, Portugal) using an ESCALAB 250Xi (Thermo Fisher Scientific) with a monochromatic Al X-ray source operating at 220 W, 14.6 kV, on a 650 μm spot size. Survey spectra were acquired at 100 eV and high-resolution C 1s spectra at 20 eV. Survey spectra analysis and deconvolution of high-resolution spectra were performed using CasaXPS 2.3.16 software. The reference of C 1s peak was set to 284.6 eV, to correct for electric charge effect. Deconvolution of high-resolution C 1s spectra was performed using the background type Shirley. The sp^2^ carbon peak was fitted using an asymmetric Lorentzian function (LF) with an asymmetry parameter of 0.14, while all other peaks were fitted using a symmetric Gaussian-Lorentzian (70:30) function. As a reference, peak ranges used were 284.2–284.5 eV for sp^2^ C=C, 284.8–285 eV for sp^3^ C-C, 285.3–286.0 eV for C-OH, 286.1–286.7 eV for C-O-C, 287.5–287.9 eV for C=O, 288.7–288.9 eV for O-C=O and 290–293 eV for π-π bonds [53,57].

### 4.3. Production of Neat PEG and PEG/GBM Composite Hydrogels

Neat PEG hydrogels and PEG/GBM composite hydrogels were produced by *in situ* crosslinking of PEG dimethacrylate (PEGDM). PEGDM powder (average M_W_ 8000 g/mol, Ref. 25428) was purchased from Polysciences (Hirschberg, Germany). Solutions with 15% *w/v* (i.e., 150 mg/mL) PEGDM containing either FLG (1% *w/v*, i.e., 10 mg/mL), FLGO (0.1, 0.5 or 1% *w/v*, i.e., 1, 5 or 10 mg/mL, respectively) or GO (0.1, 1, 2 or 4% *w/v*, i.e., 1, 10, 20 or 40 mg/mL, respectively) were prepared in distilled water. The maximum concentrations that were possible to homogeneously disperse were 1% *w/v* FLGO and 4% *w/v* GO, since the mixtures became highly viscous and difficult to homogenize, degas and handle at higher concentrations. The mixture was vortexed for 30 s and sonicated for 15 min in an ultrasonic bath (Bondelin Sonorex Digitec, 320 W) to ensure a homogeneous GBM dispersion. The redox initiators sodium metabisulfite (SMB; 97%, Sigma 161519) and ammonium persulfate (APS; 90%, Sigma 248614) were freshly prepared and added to the mixture for a final concentration of 1 mg/mL APS and 1 mg/mL SMB. The mixture was vortexed for 30 sec, quickly spun-down at low rotations to remove bubbles and finally pipetted to a mold composed of two clean slides separated by 0.54 mm thick Teflon spacers. The hydrogels films were allowed to crosslink at 40 °C for 2 h, after which they were released from the mold, soaked in distilled water and washed for 4 h in an orbital shaker (100 rpm; water renewed every hour), in order to remove unreacted macromers, oligomers and initiators’ residues. The resulting films were cut with a puncher into the desirable shape and size, depending on the experiments.

### 4.4. Mechanical Properties of Hydrogels

Tensile properties of neat and composite hydrogels were evaluated according to the Standard Test Method for Tensile Properties of Thin Plastic Sheeting ASTM D 882-02 [35]. Samples consisted of hydrated hydrogel films with 60 mm length, 15 mm width and variable thickness (measured for each condition and variable between 0.64 and 0.91 mm, depending on the swelling degree of each formulation). Uniaxial tensile tests were performed using a TA.XTplus Texture Analyser (Stable Micro Systems, Surrey, UK) with a loading cell of 5 kg. The tensile grips were initially separated by 40 mm (grasping 10.0 mm of each side of the film), the displacement rate was 0.32 mm/sec and tests were conducted until rupture. Stress–strain curves were acquired for all samples, from which the Young’s modulus (YM, indicating stiffness), ultimate tensile strength (UTS, indicating strength) and elongation at break (EB, indicating elasticity) were obtained. YM was calculated as the slope of the linear regression in the 0–0.2 mm/mm strain range; UTS was calculated as the maximum stress registered immediately before break; and EB was calculated as the correspondent strain immediately before break. 

### 4.5. Physicochemical Properties of Hydrogels

Neat PEG and PEG/GBM composite hydrogels were characterized regarding GBM dispersion in the matrix by optical microscopy, surface roughness by scanning electron microscopy (SEM), wettability by optical contact angle and swelling degree by water uptake measurements. 

#### 4.5.1. Optical Microscopy

A macroscopic image of neat and composite hydrogels was obtained using a stereomicroscope (Olympus, Tokyo, Japan) coupled with a CCD camera with a 6.3× magnification. 

The dispersion of GBM in the matrix of composite hydrogels was observed by optical microscopy. Hydrated samples of neat and composite hydrogels were imaged using the inverted fluorescence microscope Axiovert 200 (Zeiss, Jena, Germany) in brightfield mode (3 V light source, N.A. 0.55) with a 200× magnification.

#### 4.5.2. Scanning Electron Microscopy (SEM)

The surface topography of hydrogels was analyzed by SEM. Discs cut from hydrogels were vacuum-dried at 37 °C, ON. Samples were coated with Au thin films using a Leica EM ACE200 Sputter Coater. SEM was performed with a Phenom XL equipment (Phenom-World BV, Eindhoven, Netherlands) using 15 kV electron beam and under high vacuum.

#### 4.5.3. Optical Contact Angle (OCA)

The wettability of hydrated hydrogels was evaluated by OCA, which was determined by the inverted air drop method using the Data Physics goniometer model OCA 15, equipped with a CCD camera. The samples were attached to glass slides and placed in contact with distilled water. A 10 μL air bubble was released from a J-shaped needle underneath the sample’s surface, and an image was captured using the CCD camera, connected to the SCA20 software. The OCA was determined using the Young–Laplace fitting model.

#### 4.5.4. Water Uptake Measurements

Mass measurements were performed to determine the water absorbed by dried neat and composite hydrogels at specific timepoints. Small discs (Ø = 11 mm) were vacuum-dried at 37 °C, ON. Samples were pre-weighed and then immersed in distilled water. At specific timepoints, the discs were removed, blotted with filter paper (to remove excess water) and weighed. This was performed at 5, 10, 20, 30 min, 1, 2, 3, 4 and 48 h. The swelling degree was calculated as:

(1) $$Swelling\;degree\;\left(\%\right)=\frac{m_{swollen}-m_{initial}}{m_{initial}}\times 100$$

where $m_{swollen}$ is the mass of the sample at that timepoint, and $m_{initial}$ is the initial mass of the dried sample. 

### 4.6. Cytocompatibility of Hydrogels’ Extracts

In vitro cytocompatibility of neat PEG and PEG/GBM composite hydrogels was evaluated by incubating human umbilical vein endothelial cells (HUVEC) with extracts of these materials.

#### 4.6.1. Cell Line and Culture Conditions

HUVEC were thawed and cultured in gelatin-coated T-75 flasks. Complete culture medium—M199 (Sigma M4530) supplemented with 10 vol.% (0.1 mL/mL) fetal bovine serum (FBS; Gibco), 1 vol.% (0.01 mL/mL) penicillin/streptavidin (P/S; Biowest L0022, MO, USA), 90 μg/mL heparin (Sigma H3149) and 15 μg/mL endothelial cell growth supplement (ECGS; Corning 354480, NY, USA)—was refreshed every two days. When reaching approximately 80% confluency, cells were trypsinized, centrifuged (1200 rpm, 5 min), collected and counted.

#### 4.6.2. Preparation of Extracts

Neat and composite hydrogels (Ø = 11 mm) were sterilized with 70% ethanol, and rinsed with sterile phosphate-buffered saline (PBS). Extracts were prepared according to International Standard ISO 10993-12 [36], reflecting a 0.5–1.0 mm thickness and using a surface area-to-volume extraction ratio of 3 cm^2^/mL. Briefly, one 11 mm-diameter disc of each composite was incubated with 635 μL of M199 (Sigma M4530) supplemented with 10 vol.% FBS, 1 vol.% P/S and 90 μg/mL heparin. Incubation was carried out for 24 h at 37 °C, in an orbital shaker at 100 rpm. Tissue-culture polyethylene terephthalate (TCPET) was used as a reference material with no cytotoxicity. Culture medium without any material was incubated under the same conditions (herein denoted as ”24 h medium”).

#### 4.6.3. Extracts Assay

The extracts assay was performed according to ISO 10993-5 [37]. HUVEC (1 × 10^5^ cells/mL, 100 μL) were seeded on gelatin-coated TCPS wells of 96-well plates and incubated for 24 h (37 °C, 5% CO_2_). After that, the culture medium was replaced by 100 μL of materials’ extracts supplemented with 15 μg/mL ECGS immediately before incubation. Control conditions included HUVEC cultured with 24 h medium, fresh medium and medium with 0.1 vol.% Triton X-100. 

Metabolic activity was assessed at 24 h by a resazurin assay. The hydrogels’ extracts were removed, and cells were incubated with complete medium with 20 vol.% resazurin for 4 h (37 °C, 5% CO_2_). After that, the medium was collected on a 96-well plate with black walls and fluorescence (λ_excitation_: 530 nm, λ_emission_: 590 nm) and was measured using Synergy Mx (Biotek Instruments, CA, USA). 

HUVEC morphology was assessed by DAPI/phalloidin staining. Cells were fixed with 4% paraformaldehyde (PFA) for 20 min and rinsed with PBS. Permeabilization was performed with 0.1 vol.% Triton X-100 for 5 min, after which they were rinsed with PBS. Cells were then stained with phalloidin (10 μg/mL, Alexa Fluor 488, Thermo Fisher Scientific) for 30 min at room temperature (RT). Nuclei were counterstained with DAPI (3 μg/mL) for 15 min, RT, followed by PBS rinsing. Stained HUVEC were observed and imaged using the inverted fluorescence microscope Axiovert 200 (Zeiss) with a 200× magnification.

### 4.7. Anti-Adhesive Properties of Hydrogels

To understand if PEG/GBM composite hydrogels maintain the anti-adhesiveness of neat PEG hydrogels, the adhesion of HUVEC, human platelets and bacteria was studied. 

#### 4.7.1. HUVEC Adhesion Assay

Neat PEG and PEG/GBM composite hydrogels (Ø = 5 mm) were sterilized as aforementioned and placed in 96-well suspension plates. HUVEC (1 × 10^6^ cells/mL, 10 μL drop) were seeded on top of the discs and incubated for 1 h (37 °C, 5% CO_2_) to allow cell adhesion, and then 90 μL of complete medium was added. Metabolic activity was assessed at 24 h and on Day 7 by resazurin assay, as described in the previous section. HUVEC morphology was assessed by DAPI/phalloidin staining on Day 7, as aforementioned. After fluorescence staining, samples were mounted upside-down in glass coverslips using Mowiol 4-88 mounting medium and imaged using the inverted fluorescence microscope Axiovert 200 (Zeiss) with a 200× magnification.

#### 4.7.2. Platelet Adhesion Assay

Neat PEG and PEG/GBM composite hydrogels (Ø = 9 mm) were sterilized as aforementioned and placed in 48-well plates. Samples were pre-immersed in 1 vol.% human plasma solution, incubated for 1 h at 37 °C and then rinsed with PBS. Platelet concentrates were kindly provided by the Immunohemotherapy Service of Centro Hospitalar Universitário de São João, Porto. Samples were then transferred to a 48-well plate previously blocked with 10 mg/mL of bovine serum albumin (Sigma A4503), and incubated with 500 μL of platelet suspension at a concentration of 3 × 10^8^ cells/mL for 1 h at 37 °C in an orbital shaker (70 rpm). After rinsing with PBS, samples were fixed with freshly prepared 1.5 vol.% glutaraldehyde (Sigma GA00-4) in a 0.14M sodium cacodylate (Sigma 20840) buffer for 30 min, RT, and then rinsed with distilled water. Samples were dehydrated with a growing ethanol gradient, i.e., incubated with 50, 60, 70, 80, 90 and 99 vol.% ethanol solutions for 10 min each, after which they were dried with hexamethyldisilazane (Sigma H4875) ON. Platelet adhesion and morphology were assessed by SEM (as described in 2.5.3).

#### 4.7.3. Bacterial Adhesion Assay

Neat PEG and PEG/GBM composite hydrogels (Ø = 9 mm) were sterilized as aforementioned and placed in 48-well plates. The bacterial adhesion was tested using *Staphylococcus aureus* (*S. aureus*) ATCC 33591. *S. aureus* was grown in Tryptic Soy Agar (Sigma 22091) at 37 °C, ON. Two individual colonies were collected, dispersed in 5 mL of Tryptic Soy Broth (TSB; Merck 1.05459.0500, Kenilworth, NJ, USA) and cultured ON at 37 °C in an orbital shaker (150 rpm). Bacteria were centrifuged (10 min, 2700 rpm) and resuspended in 5 mL of TSB twice, and initial concentration was determined by optical density. Bacterial concentration was adjusted to 6.6 × 10^5^ cells/mL in TSB medium supplemented with 1 vol.% human plasma. Samples were placed in 48-well plates and incubated with 300 μL of this bacterial suspension for 24 h at 37 °C. After removing the medium, samples were washed with PBS, fixed, dehydrated and imaged by SEM, as previously described for platelets.

### 4.8. Statistical Analysis

Statistical analysis was performed using GraphPad Prism 7.0. Datasets were tested for Gaussian distribution using the Shapiro–Wilk normality test (α = 0.05). Unless stated otherwise, statistical differences between groups were calculated based on ordinary one-way ANOVA, followed by Tukey’s multiple comparison tests. A *p*-value lower than 0.05 was considered statistically significant, and data are shown as mean ± standard deviation (SD).

## 5. Conclusions

GBM hold great potential for the improvement of hydrogels’ mechanical properties. Oxidized forms of graphene, particularly FLGO and GO, can be used as nanofillers to increase PEG hydrogels’ tensile stiffness, strength and elasticity. The incorporation of 4% *w/v* GO causes a 6-fold increase in stiffness and 14-fold increase in tensile strength of PEG hydrogels, reaching up to 218 kPa YUTS. Significantly, this is the greatest tensile improvement reported to date for PEG hydrogels with GBM fillers. Of note, the incorporation of GBM does not alter the PEG hydrogels’ inherent non-fouling behavior towards mammalian cells, blood platelets nor bacteria. Hence, PEG/GBM composite hydrogels can be used as stiff and strong “blank slates” for multiple applications, presenting particularly interesting features for use in blood-contacting devices.

## Data Availability

Not applicable.

## Supplementary Materials

The following are available online at https://www.mdpi.com/article/10.3390/ijms23042312/s1.
