# Supplementary material for: Using Graphene-Based Materials for Stiff and Strong Poly(ethylene glycol) Hydrogels
Source: Int J Mol Sci. 2022 Feb 19;23(4):2312. doi: 10.3390/ijms23042312 (PMC8880715; doi:10.3390/ijms23042312)
Supplement: Supplementary file 1 [file ijms-23-02312-s001.zip › ijms-1583537-supplementary.pdf]

Using Graphene-Based Materials for Stiff and Strong Poly(Ethylene Glycol) Hydrogels

Helena P. Ferreira [1,2,3], Duarte Moura [1,2,4], Andreia T. Pereira [1,2], Patrícia C. Henriques [1,2], Cristina C. Barrias [1,2,3], Fernão D. Magalhães [5] and Inês C. Gonçalves [1,2,*]

[1] i3S—Instituto de Investigação e Inovação em Saúde, Universidade do Porto, Rua Alfredo Allen 208, 4200-135 Porto, Portugal; helena.ferreira@i3s.up.pt (H.P.F.); duarte.moura@i3s.up.pt (D.M.); andreia.pereira@i3s.up.pt (A.T.P.); ap.henriques@ineb.up.pt (P.C.H.); ccbarrias@ineb.up.pt (C.C.B.)
[2] INEB—Instituto de Engenharia Biomédica, Universidade do Porto, Rua Alfredo Allen 208, 4200-135 Porto, Portugal
[3] ICBAS—Instituto de Ciências Biomédicas Abel Salazar, Universidade do Porto, Rua Jorge de Viterbo Ferreira 228, 4050-313 Porto, Portugal
[4] FEUP—Faculdade de Engenharia, Departamento de Engenharia Metalúrgica e de Materiais, Universidade do Porto, Rua Dr. Roberto Frias, 4200-465 Porto, Portugal
[5] LEPABE—Laboratório de Engenharia de Processos, Ambiente, Biotecnologia e Energia, Faculdade de Engenharia da Universidade do Porto, Rua Dr. Roberto Frias, 4200-465 Porto, Portugal; fdmagalh@fe.up.pt
* Correspondence: icastro@ineb.up.pt

SUPPLEMENTARY INFORMATION

Hydrogel	PEG MW (kDa)	PEG conc. (%w/v)	Crosslinking	Incorporation of GBM	GBM conc. (%w/v)	Mechanical tests	Mechanical properties	Application	Ref.
PEGDM	0.55	70.8	Photocrosslinking<br>UV 365 nm, 15 min	rGO<br>Physical	0.079, 0.31, 0.62, 0.78, 0.92	-	-	NIR-triggered transdermal insulin delivery	[28]
PEGDM	0.7, 2, 6, 12	6, 8, 10, 12 (respectively, for each MW)	Radical crosslinking<br>APS and TEMED<br>RT, 1h	GO<br>Physical<br><br>Methacrylic-GO<br>Covalent	0.025, 0.05, 0.1, 0.2, 0.4	Compressive	For 0.4% GO composites:<br>0.7kDa: YM= 32kPa, CS= 276kPa<br>2kDa: YM= 40kPa, CS= 1000kPa<br>6kDa: YM= 36kPa, CS= 750kPa<br>12kDa: YM= 24kPa, CS= 975kPa<br>For 0.4% M-GO composites:<br>0.7kDa: YM= 20kPa, CS= 180kPa<br>2kDa: YM= 8kPa, CS= 520kPa<br>6kDa: YM= 12kPa, CS= 650kPa<br>12kDa: YM= 24kPa, CS= 975kPa	Load-bearing biomedical applications	[29]
PVA/PEG	6	13.3	Physical crosslinking (with GO)<br>Freeze-thawing, -40 °C, 8h	GO<br>Physical	0.15, 0.3, 0.6, 1.2	Tensile	Neat hydrogel:<br>TS= 20kPa, EB= 120%<br>Composite hydrogels:<br>0.15% GO: TS=100kPa, EB= 780%<br>0.3% GO: TS= 110kPa; EB= 830%<br>0.6% GO: TS= 120kPa; EB= 860%<br>1.2% GO: TS= 160kPa; EB= 920%	Skin-compatible, conductive hydrogel for ECG electrodes	[30]
PEG	1	10%	Photocrosslinking<br>Irgacure 2959, UV 365 nm, 150 sec	G-BSA<br>Physical	0.255, 0.51	AFM-enabled nanoidentation	Neat hydrogel:<br>YM= 42.6kPa<br>Composite hydrogels:<br>0.255% GO: YM= 50.1kPa<br>0.51% GO: YM= 284.2kPa	Load-bearing, conductive biomedical applications	[31]
PEG-melamine/ HA-SH	0.7	5<br>(+ 40% HA)	Chemical crosslinking<br>RT, 1 min	GO<br>Physical	0.05	Rheological	Neat hydrogel: G'= 11 Pa<br>Composite hydrogel: G'= 25 Pa	Injectable, conductive cell-laden hydrogel for cardiac repair	[32]
PEDOT:PSS/ PEGDA	0.575	20, 30, 40, 50<br>(+ 0.2, 0.4, 0.6% PEDOT:PSS)	Photocrosslinking<br>Darocur 1173, UV 365 nm, 5 sec	rGO<br>Physical	0.125, 0.25	Compressive (DMA)	Neat PEGDA hydrogel:<br>20% PEG: YM= 550kPa<br>30% PEG: YM= 1400kPa<br>40% PEG: YM= 1500kPa<br>50% PEG: YM= 1800kPa<br>Composite hydrogel:<br>0.125% rGO: YM= 230kPa<br>0.25% rGO: YM= 270kPa	Strong, conductive hydrogel for biomedical applications	[33]
GelMA/PEGDA	8	0, 2.5, 5, 10<br>(+ 5% GelMA)	Photocrosslinking<br>Ciba, UV 360 nm, 40 °C	GO<br>Physical	0.02, 0.04, 0.08	Compressive	Neat 5% GelMa/5% PEGDA hydrogels:<br>YM= 64.4kPa<br>Composite hydrogels:<br>0.02% GO: YM= 70kPa<br>0.04% GO: YM= 80kPa<br>0.08% GO: YM= 95.6kPa	Load-bearing biomedical applications	[34]

Table S1 –PEG/GBM composite hydrogels reported in literature. *Summary of formulations and mechanical testing of PEG/GBM composite hydrogels reported in literature. Abbreviations: AFM – atomic force microscopy; APS – ammonium persulfate; BSA – bovine serum albumin; CS – compressive strength; DMA – dynamic mechanical analysis; EB – elongation at break; GelMA – gelatin methacrylate; HA – hyaluronic acid; MW – molecular weight; PEDOT:PSS – poly(3,4-ethylenedioxythiophene) polystyrene sulfonate; PEGDA – PEG diacrylate; PEGDM – PEG dimethacrylate; PVA – poly(vinyl alcohol); TEMED – N,N,N',N'-tetramethylethane-1,2-diamine; TS – tensile strength; YM – Young's modulus.*

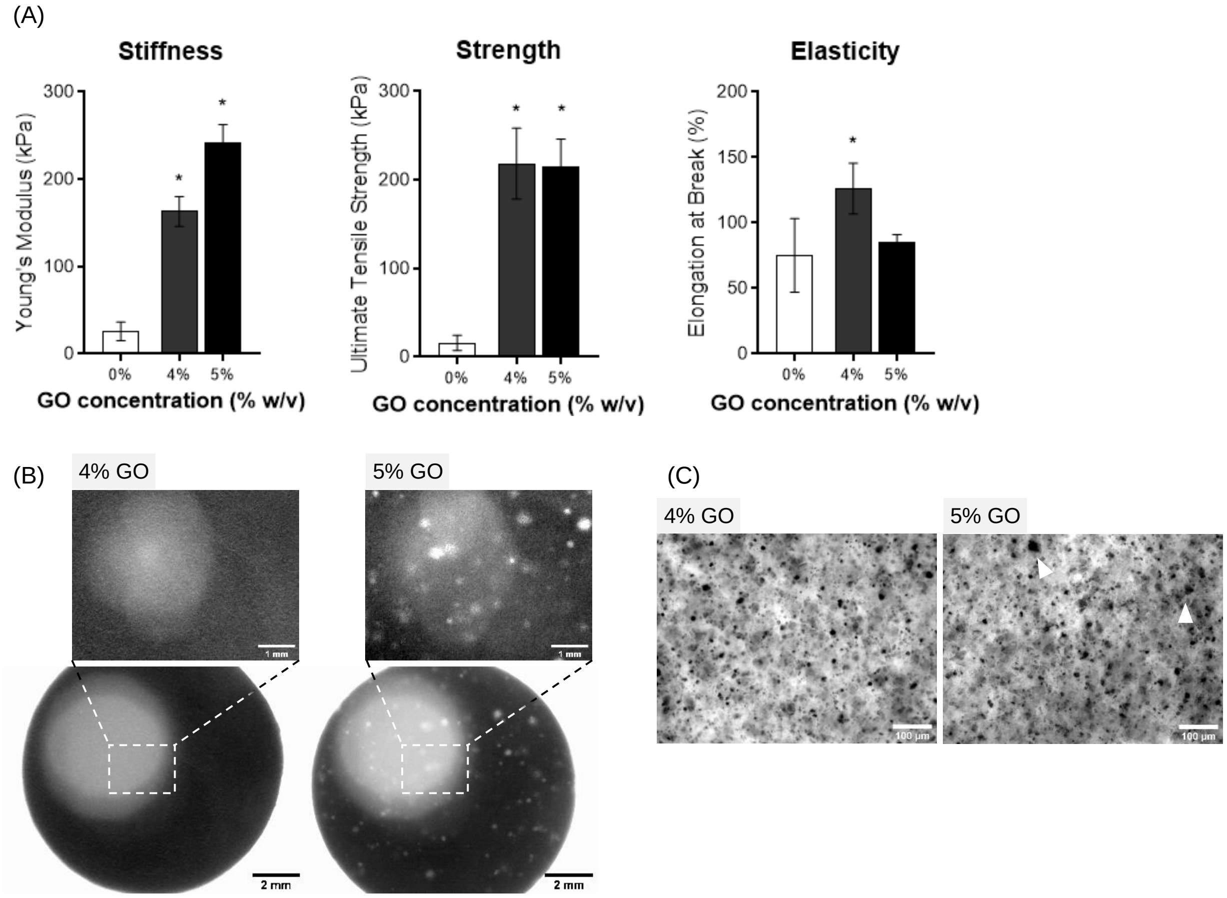

Figure S1 – Effect of GO concentration in tensile properties, macroscopic appearance and in GO dispersion in matrix of PEG/GO composite hydrogels. (A) Young's modulus, ultimate tensile strength and elongation at break of neat PEG hydrogels (0% GO), and 4% and 5% GO composite hydrogels. At least 3 technical replicates; Kruskall-Wallis analysis, Dunn's multiple comparison test, * $p< 0.05$ vs. neat PEG hydrogels; no statistically significant differences between 4% and 5% GO. (B) Stereomicroscope images of 4% and 5% GO composite hydrogels; scale bar: 2 mm; scale bar in zoomed images: 1 mm. (C) Brightfield images from widefield microscope, showing GO dispersion in hydrogel matrix; arrowheads indicate larger GO aggregates; scale bar: 100 µm.

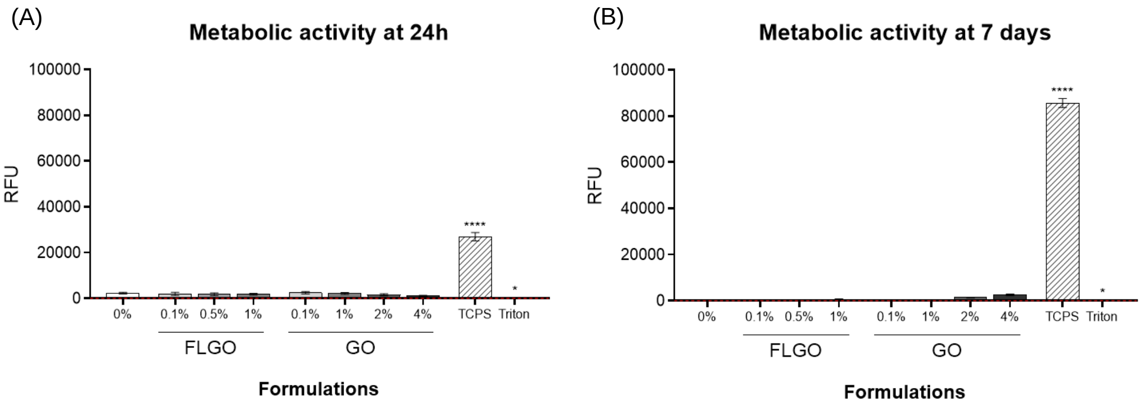

Figure S2 - Anti-adhesiveness of composite hydrogels. Metabolic activity, expressed in random fluorescence units (RFU), of HUVEC seeded on top of neat PEG, FLGO and GO composite hydrogels, gelatin-coated TCPS (positive control of cell adhesion) and incubated with 0.1% Triton X-100 (negative control of cell adhesion), after (A) 24h and (B) 7 days of culture.
